# Interweaving Knowledge Resources to Address Complex Environmental Health Challenges

**DOI:** 10.1289/ehp.1409525

**Published:** 2015-04-24

**Authors:** Beth Ellen Anderson, Marisa F. Naujokas, William A. Suk

**Affiliations:** 1 Superfund Research Program, National Institute of Environmental Health Sciences, National Institutes of Health, Department of Health and Human Services, Research Triangle Park, North Carolina, USA; 2 MDB, Inc., Durham, North Carolina, USA

## Abstract

**Background:**

Complex problems do not respect academic disciplinary boundaries. Environmental health research is complex and often moves beyond these boundaries, integrating diverse knowledge resources to solve such challenges. Here we describe an evolving paradigm for interweaving approaches that integrates widely diverse resources outside of traditional academic environments in full partnerships of mutual respect and understanding. We demonstrate that scientists, social scientists, and engineers can work with government agencies, industry, and communities to interweave their expertise into metaphorical knowledge fabrics to share understanding, resources, and enthusiasm.

**Objective:**

Our goal is to acknowledge and validate how interweaving research approaches can contribute to research-driven, solution-oriented problem solving in environmental health, and to inspire more members of the environmental health community to consider this approach.

**Discussion:**

The National Institutes of Health’s National Institute of Environmental Health Sciences Superfund Research Program (SRP), as mandated by Congress, has evolved to become a program that reaches across a wide range of knowledge resources. SRP fosters interweaving multiple knowledge resources to develop innovative multidirectional partnerships for research and training. Here we describe examples of how motivation, ideas, knowledge, and expertise from different people, institutions, and agencies can integrate to tackle challenges that can be as complex as the resources they bring to bear on it.

**Conclusions:**

By providing structure for interweaving science with its stakeholders, we are better able to leverage resources, increase potential for innovation, and proactively ensure a more fully developed spectrum of beneficial outcomes of research investments.

**Citation:**

Anderson BE, Naujokas MF, Suk WA. 2015. Interweaving knowledge resources to address complex environmental health challenges. Environ Health Perspect 123:1095–1099; http://dx.doi.org/10.1289/ehp.1409525

## Introduction

Environmental health problems arise in a wide array of locations and conditions with a broad range of potential hazards and outcomes. Addressing the diversity of challenges and factors that influence outcomes demands a diversity of knowledge resources and shared approaches. Working in full partnerships of mutual respect among wide ranges of expertise and experience can lead to multidirectional information exchange and be mutually beneficial during the research process and application of the science. For example, scientists and engineers can work with U.S. Environmental Protection Agency (EPA) risk assessors and decision makers, public health professionals, community members, and other stakeholders to mitigate hazardous chemical exposures and adverse health effects. These partnerships have the potential to maximize research impacts by more effectively and quickly translating research to help solve problems and mitigate public health risks.

The terms “interdisciplinary” and “transdisciplinary” have been used to differentiate various levels of knowledge integration and partnership (Jahn et al. 2012; Mobjörk 2010; Pohl 2011). “Community-based participatory research” is another term used to describe integrative approaches that engage community members (Minkler and Wallerstein 2010; O’Fallon and Dearry 2002). “Research translation” is also a term that can encompass integrative approaches when researchers partner with decision makers, stakeholders, and entrepreneurs to apply the science to real-life challenges (Dankwa-Mullan et al. 2010; Pennell et al. 2013). Although all of these terms describe approaches that draw on and engage resources outside of traditional academic environments, none of them alone convey the breadth of resources and depth of partnerships required in some of the more complex environmental health problems. Furthermore, “disciplinary” can imply constraints or limitations, leaving out expertise in areas that might not be considered a “discipline,” such as community members’ expertise in the details of their daily lives and culture.

Here, we use the term “interweaving” to describe how the National Institute of Environmental Health Science (NIEHS) Superfund Research Program (SRP) has incorporated all of these approaches and a very wide range of knowledge resources in both formal and informal multidirectional partnerships toward research-driven, solution-oriented activities. The widely diverse resources are woven together to create a knowledge fabric that is permeable, flexible, adaptive, and without hierarchy of importance or value. Resources form the warp and weft of the fabric, weaving together to increase the tensile strength and build capacity to improve public health. This fabric is strengthened as distinct borders of contributing resources blur, partnerships are built, and resources are shared. Motivation, ideas, knowledge, resources, and enthusiasm from different people, institutions, and agencies weave together to tackle problems that can be just as complex as the resources they bring to bear. The diverse resources are like the parts of a quilt that are layered in multiple dimensions with different fabric types and textures. Our intent in introducing the term “interweaving” is to move away from restrictions and preconceptions associated with conventional terms to highlight the potential of using diverse knowledge resources, perhaps more diverse than what conventional terms imply.

Integrating science with its user community is a recognized mechanism for conducting environmental health research, demonstrated by NIEHS programs such as the Breast Cancer and the Environment Research Program, Partnerships for Environmental Public Health, and the SRP (NIEHS 2015a). What makes the SRP arguably unique is the very wide range of knowledge resources—from geoscientists to community members—that is brought together in full multidirectional partnerships to solve real-life problems and reduce disease burdens. Our goals for this paper are to acknowledge and validate how interweaving research approaches can contribute to research-driven, solution-oriented problem solving in environmental health, and inspire more members of the environmental health community to consider this approach.

## Discussion

*Interweaving widely diverse knowledge resources.* The NIEHS Superfund Hazardous Substance Research and Training Program, commonly called the Superfund Research Program (SRP), actively incorporates an interwoven approach in its large multiproject research grants. The breadth and depth of expertise within the SRP framework spans the spectrum from engineers and scientists to government, community, and industry partners. Specific SRP examples described here demonstrate that partnership formation in problem solving does work. Additional project descriptions of other SRP grantees’ integrative work are described elsewhere (NIEHS 2015c). The interweaving approaches used by the SRP provide a way of thinking about how to do science and use science to solve environmental health problems. After decades of fostering these approaches, the SRP has seen successes with demonstrated enrichment of the research process. Researchers strengthen their inquiries, put science to use, and build capacity and scientific understanding outside of the traditional academic community.

Here, we highlight examples that often are not seen in the environmental health research community because the stories are spread among journals that span the diverse disciplines that they represent. Furthermore, some of the stories are not published in conventional professional journals; instead, websites, brochures, and unpublished activities that benefit their intended audience serve as evidence of their work (CES4Health 2015). We are, in effect, converging information resources to describe interweaving projects that might otherwise go unnoticed by many researchers in the environmental health community. Without a doubt, incorporating knowledge resources outside of traditional academics enriches the research process and makes SRP a richer, more effective research program. Here we illustrate how the SRP is a functioning model that encourages researchers to consider reaching beyond conventional knowledge resources to engage with diverse partners and unlock the potential of achieving more than would be achieved through other approaches.

*The SRP research and engagement framework.* The SRP was created more than 25 years ago by a Congressional mandate in the Comprehensive Environmental Response, Compensation, and Liability Act (CERCLA) as amended by the Superfund Amendments and Reauthorization Act of 1986 (SARA 1986). At the time the SRP was created, the Congressional mandate to include a wide range of disciplines within one research program was quite novel and presented a challenge in a climate where universities were department-oriented in a silo-type structure. The mandate to find realistic ways to solve environmental health problems required a new approach to environmental health research.

From its inception, the SRP has emphasized a strong foundation of high-caliber basic science research and training, which continues to this day. Growing from that ever-present foundation, SRP’s integrative framework has challenged the norms in research and training. In 1999, SRP published a unifying framework for multidisciplinary research that illustrated interrelatedness among human, ecological, and remediation research (Suk et al. 1999). The framework evolved over time, and now the SRP functions more like a boundary organization (Crona and Parker 2011; Guston 2010). Whereas boundary organizations are often associated with work at science/policy interfaces, the SRP traverses a broad range of scientific, engineering, sociological, community, government, and industry expertise. The evolution of the SRP framework reflects what some scholars see as a shifting knowledge landscape that calls for more development of these approaches in research environments (Dankwa-Mullan et al. 2010; Russell et al. 2008; Vasbinder et al. 2010).

Explicit in its design of fostering integration, the SRP has long encouraged and now requires formation of Community Engagement Cores (CECs) that facilitate community involvement with researchers. Each SRP Research Center also has a Research Translation Core (RTC), whose task is to maintain effective communication between diverse partners inside and outside of academic environments and facilitate the application of SRP scientific accomplishments (NIEHS 2015c). Successful RTC and CEC projects result from building relationships and understanding, which then enables effective interactions, brainstorming, information gathering and dissemination, new tool implementation, and, most importantly, problem solving. The SRP also fosters interweaving at the macro level by bringing together RTCs and CECs from different research centers at annual meetings, regional meetings, monthly conference calls, intercenter working groups, and informal gatherings at other national meetings.

*Examples of academic/community partnerships.* The University of Arizona (UA) SRP approach at the Iron King Mine and Humboldt Smelter Superfund Site (IKMHSS) in Dewey-Humboldt, Arizona is a good example of interweaving resources focused on real-world problems. Mining wastes known as mine tailings remain in the area in piles of dust and soil, covering more than 150 acres that contain high concentrations of metals and other contaminants. Concerns at the site are focused primarily on arsenic and lead contamination of groundwater, surface water, air, soil, and house dust (Solís-Dominguez et al. 2012). To address these concerns at the IKMHSS, the UA SRP takes an approach that includes basic science research, environmental engineering, risk assessment, and community education and engagement (Ramirez-Andreotta et al. 2014b). The benefits are multidirectional in terms of understanding, capacity building, and problem solving.

For example, the UA SRP engaged with local residents to start a research project called Gardenroots, which used a community-based participatory research type of approach with components of citizen science and community capacity building. Residents planted gardens and collected samples for UA SRP testing and risk assessment analysis. Arsenic concentrations varied among different types of vegetables, and exposure assessment modeling revealed that dietary contribution to total arsenic exposure was small compared with water and soil (Ramirez-Andreotta et al. 2013). The UA SRP reported results to the community and provided suggestions for reducing exposures (Ramirez-Andreotta 2013). As a result, citizen participants gained a richer understanding of soil contamination, exposure prevention, relative risks, and the scientific process. Furthermore, citizens learned how to gather information and take appropriate action, demonstrated recently when community members effectively notified authorities when citizens’ water testing revealed elevated arsenic levels (Ramirez-Andreotta et al. 2014a). Interweaving SRP researchers with community continues to provide multidirectional exchange of expertise and resources.

Another SRP Research Center, at Brown University, uses interweaving approaches in several projects (Brown et al. 2012; Cohen 2010; Senier et al. 2008), including a long-standing project that has focused on school siting decisions in Providence, Rhode Island (Brown P, personal communication). One example focused on the Reservoir Triangle neighborhood where Alvarez High School was built on the former Gorham Silver Manufacturing Company site, a site that is highly contaminated with multiple toxicants including trichloroethylene (TCE) and potentially toxic metals. The Rhode Island Department of Environmental Management raised concerns about vapor intrusion in schools, and the story garnered publicity in the local press. Brown University SRP researchers, with extensive knowledge about vapor intrusion (Yao et al. 2013), were eager to get involved. Brown University SRP scientists and their CEC colleagues partnered with the Rhode Island Department of Environmental Management staff, the U.S. EPA, the Environmental Justice League of Rhode Island, and other community partners and activists. The results informed efforts that were instrumental in passage of legislation (R.I. Gen. Law 23-19.14-4), which set specific regulations in school siting in Rhode Island, including prohibition of building or expanding on vapor intrusion sites (Environmental Justice League of Rhode Island 2013). The Brown University SRP benefitted on several levels: Productive partnerships on a high-profile issue brought positive publicity to Brown University SRP; the partnerships forged relationships and extended networks for all involved; and participants gained personal satisfaction in knowing that they made a difference in citizens’ lives (Brown 2013). Most important, the Brown University SRP translated scientific knowledge to policy outcomes.

*Examples of academic/tribal partnerships.* Fully engaged integrative partnerships between tribal nations and academic institutions present unique cultural, legal, and communication challenges (Harding et al. 2012b). The Oregon State University (OSU) SRP and members of the Confederated Tribes of the Umatilla Indian Reservation (CTUIR) Environmental Health Program have nurtured a successful collaboration by investing substantial efforts into enhancing cultural and scientific knowledge as well as sensitivity between academic and tribal researchers and community advocates (Harding et al. 2012a; Oregon State University 2015; Schure et al. 2013). CTUIR scientists sought OSU’s expertise in polyaromatic hydrocarbon exposures and toxicity out of concern for exposures related to traditional tribal activities such as salmon-smoking methods. OSU sought to learn more about tribal customs of daily living to better inform exposure and risk assessments. The OSU and CTUIR mutually embedded staff in both settings, OSU and tribal laboratories, with a shared goal of maintaining tribal cultural heritage while protecting tribal public health. Unique sovereignty, research ethics, data sharing, intellectual property, and informed consent issues are addressed in specific written agreements that are signed by all researchers and study participants, helping to build trust that sustains the partnership (Harding et al. 2012b). Through their partnership, researchers developed exposure scenarios that reflect tribal-specific patterns of traditional subsistence “lifeways” (Forsberg et al. 2012; Harper et al. 2012). The interweaving approach resulted in greater research capacity for both the OSU and CTUIR, and their cultural and scientific insights may be applied to future exposure and risk assessments in tribal populations (Harper et al. 2012).

*Examples of academic/government partnerships.* The interweaving approach is also applied to SRP work with government agencies. Integrative partnerships with the U.S. EPA, Agency for Toxic Substances and Disease Registry (ATSDR), state agencies, and scientific advisory boards can result in better-informed toxicity research, risk assessments, and regulatory decision making. One example is the SRP Research to Risk Assessment (R2RA) Project (NIEHS 2015b). The goal of the R2RA is to create a network of interagency relationships for ongoing collaboration among SRP researchers and key senior staff partners from the SRP, U.S. EPA, ASTDR, and National Toxicology Program (NTP) to better define research needs and promote use of cutting-edge research in risk assessments. One R2RA pilot project brings together a senior risk assessor from U.S. EPA Region 2 with researchers from the U.S. EPA Office of Research and Development, NTP, and the University of Iowa (UI) SRP to tackle problems related to airborne polychlorinated biphenyls (PCBs) in New York City public schools. The U.S. EPA currently does not have an inhalation reference concentration for PCBs for gauging potential health risks from inhalation, and there is little data available for this route of exposure. UI SRP researchers with a strong background in airborne PCB research (Dhakal et al. 2013) are working with these risk assessors and toxicologists to design experiments specifically to inform PCB inhalation risk assessments. These types of studies can be expensive, and by planning experiments in a collaborative effort, experiments are more likely to be productive and cost effective, while providing valuable information for U.S. EPA risk assessments (Maddaloni M, personal communication).

*Examples of academic/public health partnerships.* SRP Research Centers also actively engage with public health professionals to help address problems in their communities. The University of North Carolina (UNC) SRP RTC has been working with the North Carolina Department of Health and Human Services (NCDHHS) on several projects with the goal of enhancing NCDHHS capacity to protect public health. The NCDHHS was accumulating data on concentrations in private well water for 31 contaminants of concern for more than 63,000 wells, but did not have the capacity to analyze the data. The UNC SRP brought analytical tools and expertise to geospatially code data and generate maps of arsenic and TCE concentrations. The geocoding process resulted in a more than 10-fold increase in knowledge of contaminant locations in the state, and additional mathematical modeling studies predicted “hot spots” in unmonitored areas with increased likelihood of high arsenic concentrations (Sanders et al. 2012). The RTC continues to work with the NCDHHS to inform residents about possible well water contamination. This integrative project engaged the wide-ranging expertise of state and county public health officials, a state toxicologist, and county public health educators, together with SRP statisticians, environmental health scientists, chemists, and information technology experts. The partnerships led to the development of decision support and analytical tools, as well as public health strategies to address concerns related to TCE exposures (Gray 2010).

*Interweaving approaches and career development.* The SRP views integrative training and career development as key elements for bridging cultural divides and divergent vocabulary outside of traditional academic environments. From this perspective, the SRP fosters agility in working with multiple partners across disciplines and interests. SRP-funded graduate-level course topics include environmental health science, policy, and law (Boston University SRP) as well as environmental justice related to Superfund sites (UNC SRP). The University of Kentucky SRP trainee workshops teach graduate students and postdoctoral scholars to tailor research presentations for community and policy audiences with varying scientific literacy (Hoover A, personal communication). At the University of California Davis SRP, the School of Management offers an entrepreneurship training course covering business community thought processes and skills to facilitate commercialization of scientific discoveries and advance application of the science (Spier C, personal communication). At the Brown University SRP, integrative training combines sociology and anthropology with engineering and chemistry in trainees’ research projects. Upon graduation, several students obtained joint appointments across disciplines (e.g., sociology and environmental health), indicating the commitment to integrative expertise that has been nurtured (Brown 2013).

Once trainees experience integrative partnership experiences, they often seek more opportunities (Brown P and Maier R, personal communication). UA SRP trainee Michael Stovern described the process as scientists “putting themselves out there” and planting seeds for partnerships that strengthen the research while trainees can learn skills that translate to the private sector (Stovern M, personal communication). Juliana Gil-Loaiza, Corin Hammond, and Christopher Olivares agreed that SRP training experiences outside of the lab provide opportunities to expand networking, to see and feel the impacts of their research, and to stay in touch with real-world situations (Gil-Loaiza J, Hammond C, and Olivares C, personal communication).

As trainees become junior faculty, it is important to support their continuing efforts by giving credit for interweaving research activities as part of their academic portfolio. Senior faculty need to consider publications in journals or other sources outside their primary field of expertise as valued scholarship for career advancement consideration (Brown 2013). To provide assessment measures for such consideration, integrative research partners can use tangible metrics for qualitative studies and other activities to measure effectiveness and outcomes (Brown et al. 2012; Drew et al. 2012; Klein 2008; Roux et al. 2010). As interweaving research approaches become more common, integrative measures of project and career success will also become more important.

*Personal perspectives of experiences with integrative approaches.* Positive outcomes of interweaving approaches are evident in specific projects, such as the examples provided here. There are also personal benefits for involved researchers and engineers. In personal conversations, SRP grantees who are engaged in these approaches have said that input from community members and agencies, either as questions or as information, often provides new perspectives and informs innovative research approaches. Some grantees said that the innovation and potential for practical outcomes increases their chances for funding success, and the new networking supports their career and opens doors to new career directions. Many grantees enjoy seeing the practical application of their work as well as the benefits to humanity immediately and long-term.

Researchers also described challenges in doing this type of work. Building trust and understanding between partners was a common challenge. In the OSU SRP/CTUIR tribal partnership, cultural differences were overcome over the course of several years (Harding et al. 2012a). One of the tribal partners, Barbara Harper, described their current relationship using an analogy to national embassies. The ambassador visits, but it is the embassy staff that live and work with the local citizens, who understand their thoughts and concerns (Harper B, personal communication). By embedding staff in both camps, she said they reached a cultural understanding over time. Anna Harding, at the OSU SRP said, “It’s easy for university faculty to say that they engage in equal partnerships with communities, but this involves a culture change on the part of academics, and a lot of hard work with the community partners (in this case the Tribes) to also hold up their end of the bargain so the research can get done” (Harding A, personal communication).

As a result of its partnership building, OSU modified research plans in order to address tribal concerns and at the same time gather valuable scientific information that they might not have gathered otherwise. For example, the OSU CEC (which includes members from their tribal partners) measured polycyclic aromatic hydrocarbon (PAH) exposures of tribal members who were engaged in the traditional smoking of salmon (Motorykin et al. 2015b). After seeing results of the study, tribal members expressed a desire to understand the contribution of traditionally smoked salmon to personal exposure in the context of other sources of PAHs in the community. The CEC then developed a smoked fish metabolism study with tribal participants, and determined the types of PAHS that were created, absorbed, and excreted in the human body after eating traditionally smoked salmon (Motorykin et al. 2015a). Changing the research process as a result of interweaving partnerships is an important and valuable part of interweaving approaches.

## Conclusions

Interweaving approaches encourage all partners to leverage resources with increased potential for innovation and problem solving, and these approaches can work. Diverse knowledge resources form the warp and weft of the fabric, weaving together to increase the tensile strength and build capacity to improve public health. Although some of the translation of knowledge and benefits of the research may still eventually occur without these approaches, with them we are better able to proactively expedite the transfer of knowledge and a more fully developed spectrum of human health beneficial outcomes of research investments. The publishing community should consider ways to help disseminate interweaving projects’ work products and findings in conventional journals to reach across disciplines to encourage this approach. Potential integrative partners—like any of the diverse partners described here—should consider stepping outside of their conventional circle of knowledge resources to tap into the hidden riches of interweaving approaches to solve complex environmental health problems.
